# Women, Water Management, and Health^1^

**DOI:** 10.3201/eid1011.040237

**Published:** 2004-11

**Authors:** Susan Watts

**Affiliations:** * American University in Cairo, Cairo, Egypt

**Keywords:** women, water, Egypt, schistosomiasis, diarrhea, trachoma, commentary

Women play a major role in domestic water management in areas where safe water and drainage are not available in the house. In these settings, women are typically responsible for collecting, storing, and using water and for disposing of wastewater (*1**,**2*). Most studies of women's water management and the health benefits of safe water and sanitation examine the effect of protected water sources, such as covered wells or pumps, and basic sanitation (*3*). However, water management may also be a health issue in large villages and periurban communities that are supplied with piped water but have inadequate sanitation or drainage facilities. For example, in Egypt's Nile Delta, tap water is available in most rural communities (although not in every house), and no absolute shortage of water exists. However, safely disposing of wastewater and toilet effluent often remains a problem; this problem is exacerbated by the high water table associated with the irrigation system.

We conducted a study on *Schistosoma mansoni* in two Nile Delta villages (each with a population of ≈8,000) from 1991 to 1998. During this period, villagers risked infection with *S. mansoni* when they came into contact with water in irrigation canals; women were especially at risk when washing laundry and utensils in the canal.

In our 1992 survey, both study villages had access to piped water; 78% of households in al-Garda and 39% in al-Salamuniya had household connections. Al-Garda village had a pipeborne sewage system, but only one third of households were connected to it. Although 98% of households in al-Garda and 94% in al-Salamuniya had toilets, many of those households not connected to the sewage system did not safely dispose of effluent. In some cases, effluent in sewage vaults contaminated the subsoil water, and 25% of the toilets in al-Garda and 65% of these in al-Salamuniya had to be emptied periodically, usually with a bucket. A few toilets in both villages illegally emptied directly into a canal or drain. Unsafe disposal of latrine effluent was implicated in schistosomiasis transmission.

Examining water management in these two villages, we asked what choices women had and why they made decisions that continued to expose them to the risk for schistosomiasis. They found that advice to "Keep away from the canal!" was not relevant to their situation. A number of factors influenced women's water management choices, and hence, their use of the canal: effort involved, water quality and cost, and an appreciation of the opportunity for social interaction with relatives and neighbors.

Women washed domestic utensils and clothes at the canal because it saved them time and effort. If they had no household connection, they had to carry water into the house from a public standpipe. Even in households with an inside tap, women often stored water in case the supply was interrupted. For most women, those who lived in houses without a drainage system, the biggest problem was that all water used in the house had to be carried outside and thrown into the street or canal.

Water quality was also an issue. Women recognized that water from the piped (subsurface) supply was "hard" compared to canal water; in al-Garda, water hardness in the canal measured 210–280 mg/L, compared to 450 mg/L for water from the piped system. Washing at the canal used less soap, and laundry and utensils looked cleaner than if they were washed at home.

A few women said that they used the canal because of the increased cost of water. In the early 1990s, the national water authority was phasing out public standpipes, installing metered connections to every household, and charging for water according to the amount used, rather than a flat rate. Within a few months, the cost of water per cubic meter had risen 250% (*4*).

Our study suggests that the potential health benefit of piped water supplied to each house is limited if no way to remove wastewater exists. Under recent "cost recovery" policies, poor families may not be able to pay the bills for water, drainage, and sanitation; nor can they pay the full costs required to link up to such systems. Less expensive alternatives are essential; otherwise many households will go without safe drainage and sanitation.

The recent article by Clasen and Cairncross (*5*) indicates that water management is a high-profile health issue. The authors focus their discussion of the effect of water management on diarrheal diseases. Globally, these kill an estimated 2.5 million people a year, largely among children younger than 5 years (diarrheal diseases are the second most important cause of death among infants in Egypt). The authors do not directly identify water management as a gender issue, but they do point out that epidemiologic studies rarely look at water quality at the point of use. The process of storing and using household water has considerable risk for microbial contamination, even if the water comes from treated, piped sources, and is usually a woman's responsibility (*6*).

In Egypt, as in poor countries throughout the world, the availability of safe sanitation, especially in rural areas, lags behind that of safe water (*7*). Epidemiologic studies have indicated that safe sanitation may be even more important than safe water to reduce diarrhea death rates (*3*). As a health issue, then, safe water and safe sanitation are indissolubly linked.

Three hygiene-related behaviors protect infants against diarrhea: washing hands before preparing food and after using the toilet, safely disposing of infant feces, and safely storing water in the house (*5*). In Egypt and elsewhere, we need to identify constraints facing rural women that prevent them from adopting these protective behaviors. Identifying barriers to women's adopting certain hygiene, water, and sanitation behaviors could also be important for other health concerns, such as trachoma, recently recognized as resurgent in rural Egypt (*8*).

These examples suggest that in planning effective control strategies for diseases associated with a lack of safe water and sanitation, we need a greater understanding of women's water management and hygiene behaviors and local constraints they experience. We need to incorporate this knowledge into health promotion, including behavior change. Another issue is women's empowerment, strengthening their ability to make their concerns heard within the community and beyond. Expecting women to change their behavior is unrealistic unless water quality and, especially, drainage and sanitation are upgraded. Policy for safe water and sanitation needs to ensure that water is regularly accessible and safe at the point of use and that sewage and wastewater can be disposed of safely. Above all, a commitment is needed from national governments to provide and maintain safe and affordable water and sanitation for all citizens.
